# Pharyngeal Electrical Stimulation for Treatment of Poststroke Dysphagia: Individual Patient Data Meta-Analysis of Randomised Controlled Trials

**DOI:** 10.1155/2015/429053

**Published:** 2015-11-24

**Authors:** Polly Scutt, Han S. Lee, Shaheen Hamdy, Philip M. Bath

**Affiliations:** ^1^Stroke, Division of Clinical Neuroscience, University of Nottingham, Nottingham NG5 1PB, UK; ^2^Centre for Gastrointestinal Sciences, University of Manchester, Salford M6 8HD, UK

## Abstract

*Background*. Dysphagia after stroke is common, associated independently with poor outcome, and has limited treatment options. Pharyngeal electrical stimulation (PES) is a novel treatment being evaluated for treatment of poststroke dysphagia.* Methods*. We searched electronically for randomised controlled trials of PES in dysphagic patients within 3 months of stroke. Individual patient data were analysed using regression, adjusted for trial, age, severity, and baseline score. The coprimary outcomes were radiological aspiration (penetration aspiration score, PAS) and clinical dysphagia (dysphagia severity rating scale, DSRS) at 2 weeks; secondary outcomes included functional outcome, death, and length of stay in hospital.* Results*. Three completed trials were identified: 73 patients, age 72 (12) years, severity (NIHSS) 11 (6), DSRS 6.7 (4.3), mean PAS 4.3 (1.8). Compared with no/sham stimulation, PES was associated with lower PAS, 3.4 (1.7) versus 4.1 (1.7), mean difference −0.9 (*p* = 0.020), and lower DSRS, 3.5 (3.8) versus 4.9 (4.4), mean difference −1.7 (*p* = 0.040). Length of stay in hospital tended to be shorter: 50.2 (25.3) versus 71.2 (60.4) days (*p* = 0.11). Functional outcome and death did not differ between treatment groups.* Conclusions*. PES was associated with less radiological aspiration and clinical dysphagia and possibly reduced length of stay in hospital across three small trials.

## 1. Introduction

Stroke is the main cause of adult disability and the third most common cause of death in Europe. Acute stroke is complicated by oropharyngeal dysphagia in up to 50% of patients and although it often resolves over the following weeks, 40% of these patients can remain dysphagic a year later [1]. Dysphagia leads to aspiration and a 3-fold increase in pneumonia and malnutrition [2]. Patients who remain chronically dysphagic require enteral feeding through a nasogastric tube (NG) or percutaneous endoscopically introduced gastrostomy tube (PEG) and are more likely to require long-term institutional care [3]. Although dysphagia may be treated by a number of techniques, there are no definitive interventions [4].

Human swallowing has bilateral representation in the cerebral cortex but commonly with a dominant hemisphere (which is unrelated to handedness) [5]. Dysphagia has been shown to often follow a stroke that affects the dominant cortex [6] or after a recurrent stroke. Importantly, swallowing is highly dependent on afferent feedback from bulbar cranial nerves innervating the upper aerodigestive tract and a number of reports have demonstrated that increased sensory input can drive long-term beneficial changes in the cortical control of swallowing [7], and this is associated with functionally relevant reorganisation of the swallowing cortex [6, 7].

In normal volunteers and patients with subacute stroke and dysphagia, pharyngeal electrical stimulation (PES) at 5 Hz and 75% of maximum tolerated intensity (typically ~10–20 mA) for 10 minutes produced the strongest effect on brain excitability measured with transcranial magnetic stimulation (TMS) [8]. Similar stimulation was able to completely reverse a virtual lesion induced in the pharyngeal motor cortex (by slow frequency repetitive TMS) in healthy subjects [8]. A similar treatment paradigm was also most effective in dysphagic patients after stroke in a dose comparison study [9]. In a sham-controlled randomised phase II trial, PES was associated with a reduction in dysphagia, assessed using the dysphagia severity rating scale (DSRS) (see below), aspiration, and length of stay in hospital [9]. In a further phase II randomised sham-controlled trial, PES was associated with nonsignificant trends to less clinical dysphagia, more removal of nasogastric tube, and a shorter length of stay. In all of these studies, PES was safe and well tolerated [9, 10].

The aim of the present study was to assess the safety and efficacy of PES on radiological aspiration and clinical dysphagia using individual patient data from the completed randomised controlled trials. Aggregation of data from all available trials reduces the influence of any one study, better defines the point estimates of effects, and reduces the range of possible effect sizes. A secondary aim was to assess optimal methods for analysing aspiration and dysphagia scores in patients with stroke.

## 2. Methods

### 2.1. Ethics

The anonymised individual patients data come from randomised controlled trials that each involved obtaining approval from national and local research ethics committees and written informed consent from the patients or proxy consent from a designated person (partner, close family, or close friend), as appropriate. Hence, no research ethics approval or specific consent was needed for this study.

### 2.2. Search for Trials

Completed randomised controlled trials that investigated the effect of PES versus no/sham PES in patients with recent stroke and dysphagia were sought with searches (November 2014) of electronic databases including Cochrane Library (issue 3 2014), EMBASE, MEDLINE, and Science Citation Index (ISI Web of Science). Reference lists from identified reviews and trial publications were also checked for additional trials. When duplicate publications were identified, data from the primary report were used. Publications could be in any language.

### 2.3. Relevant Trials

Trials were included if they were published, enrolled adults with ischaemic stroke or haemorrhagic stroke within 90 days of onset, and involved the randomised delivery of pharyngeal electrical stimulation versus control (sham or open-label) treatment. Studies were excluded because they either were not a randomised controlled trial, were ongoing, did not include patients with stroke, included patients with ventilated or chronic stroke, or did not measure relevant outcomes.

### 2.4. Outcomes

The primary outcome was radiological aspiration as assessed using the 8-level ordinal penetration aspiration score (PAS, by videofluoroscopy) at about 2 weeks after randomisation. The PAS score ranges from 1 (material does not enter the airway) to 8 (aspiration without either a reflexive or conscious attempt to expel material) [13]. Aspiration was defined as PAS >3* a priori*; although this is different from the definition of PAS >5 as used by Rosenbek and colleagues [13], scores of 4–8 signify aspiration (PAS 6–8) or material remaining at the level of the vocal folds (PAS 4 or 5). Clinical dysphagia at 2 weeks was assessed using the dysphagia severity rating scale (DSRS) [9], a derivative of the dysphagia outcome and severity scale [14]. The DSRS ranges from 0 (normal fluids, normal diet, and eating independently) to 12 (no oral fluids, no oral feeding) [9]. Clinical dysphagia was defined* a priori* as DSRS >3 and severe dysphagia as DSRS >7.

Secondary outcomes included severity/impairment (National Institutes of Health Stroke Scale, NIHSS [15]), respiratory tract infection (including pneumonia, a key complication of dysphagia), length of stay in hospital (a key early health economic measure), and death.

### 2.5. Data Sharing

The chief investigator of each identified study was approached to join the collaboration and share individual patient data. Data were transferred electronically in statistical programme or Excel file formats. Data were then imported into a single database. Before analysing the database as a whole, representative analyses were performed for each trial to ensure that results matched those that had been published.

### 2.6. Statistics

All analyses were by intention to treat. Since death is a common outcome after stroke in patients with dysphagia, and to avoid missing an effect whereby a treatment might improve dysphagia but be unsafe, an extreme value was added to the outcome aspiration and dysphagia scores, as is done routinely for the modified Rankin Scale (mRS) measure of dependency after stroke where death is assigned a value of 6 [16]; therefore on-treatment PES ranged from 1 to 9 (where death = 9) and DSRS ranged from 0 to 13 (death = 13). The primary analysis of PAS used the mean score across 6 boli; secondary analyses of PAS were also performed to assist in determining which approach might be optimal in future studies and these assessed mean score across 3 boli, the cumulative PAS score across 6 boli (as used in the source trials [9, 10]), number of boli with a PAS score >3, number of patients with any bolus >3, and worst PAS score across all boli. The effect of PES on PAS (mean score across 6 boli) was assessed in prespecified subgroups including age, sex, stroke syndrome [12], severity, severe dysphagia (DSRS >7), aspiration (mean PAS >3), number of PAS scores >3, trial, stroke type (ischaemic, intracerebral haemorrhage), and stimulation current. The primary analysis of DSRS used the mean of patients scores; secondary analysis assessed the number of patients with DSRS score >3 and the number of patients with DSRS score >7. The effect of PES on DSRS was assessed in the same prespecified subgroups as used for PAS.

Statistical analyses used binary logistic regression, ordinal logistic regression, multiple regression, with adjustment for age, relevant baseline score, impairment (NIHSS), and trial; unadjusted analyses were also performed for completeness (where the analysis was only adjusted for trial). Data are number (%), median [interquartile range], or mean (standard deviation). Effect size is presented as odds ratio or mean difference, with 95% confidence intervals (95% CI). Analyses were performed using SAS version 9.3 and *p* < 0.05 is considered significant.

## 3. Results

### 3.1. Included Trials

Searches identified 22 studies of which 19 studies were excluded because they either were ongoing, did not include relevant outcomes [8], or were not relevant for other reasons (Supplemental Table I in Supplementary Material available online at http://dx.doi.org/10.1155/2015/429053). Three completed and published trials were identified that assessed PES in patients with recent stroke and dysphagia (Supplemental Figure I); key design and protocol criteria are given for these in Table 1. One study was a small single centre phase II trial and used a dose comparison design [9]; only data from the group that were stimulated at 5 Hz for 10 minutes over 3 days were included so that the stimulation parameters match those used in the other trials; the control group were used whereas data from active groups having other stimulation paradigms were not included. The other two trials were multicentre and parallel group in design [9, 10]. In the latter study, 17 of 35 participants had a valid VFS at baseline, and 14 had a valid VFS at 2 weeks [10]. All three studies were sponsored by an academic institution (University of Manchester).

### 3.2. Enrolled Patients

Altogether, the three trials recruited a total of 73 patients with 37 randomised to PES and 36 to no/sham PES (Table 2). The average age was 72.0 (11.8) years with 45 (61.6%) being male. Women had higher point estimates for severity markers of stroke, aspiration, and dysphagia than men but none of the differences were significant: NIHSS: women 11.5 (6.3), men 10.0 (5.5) (*p* = 0.28); PAS: women 4.3 (1.6), men 4.2 (2.0) (*p* = 0.80); DSRS: women 7.7 (4.3), men 6.2 (4.2) (*p* = 0.20). The mean time from stroke admission to randomisation was 15.1 (8.5) days. Thirty-six (49.3%) patients were taking food enterally at enrolment. Sixty-nine (94.5%) of patients had an ischaemic stroke; patients with ischaemic and haemorrhagic (ICH) stroke did not differ in severity: NIHSS 10.4 (5.9) versus 12.5 (3.1), difference 2.1 (95% CI −3.9, +8.0), and *p* = 0.50. The mean PAS and DSRS were 4.3 (1.8) and 6.7 (4.3), respectively, at baseline (Table 2).

### 3.3. Stimulation Levels

The mean threshold sensitivity was 11.4 (5.6) mA and treatment level was 16.8 (6.6) mA (Table 2).

### 3.4. Radiological Outcomes

**Figure 1:**
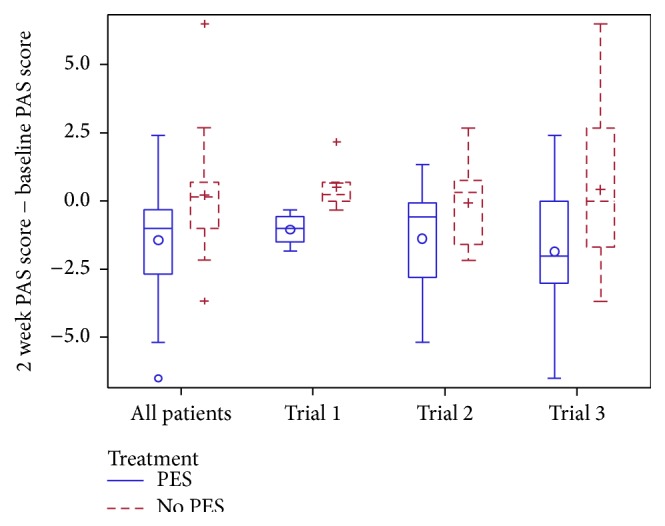
Box and whisker plot of change in penetration aspiration score from baseline to two weeks, by source trial. Comparison of pharyngeal electrical stimulation (PES) versus no PES by multiple linear regression with adjustment, overall mean difference −0.9 (95% confidence interval −1.7, −0.1; *p* = 0.020); no difference between trials (*p* = 0.89).

**Figure 2:**
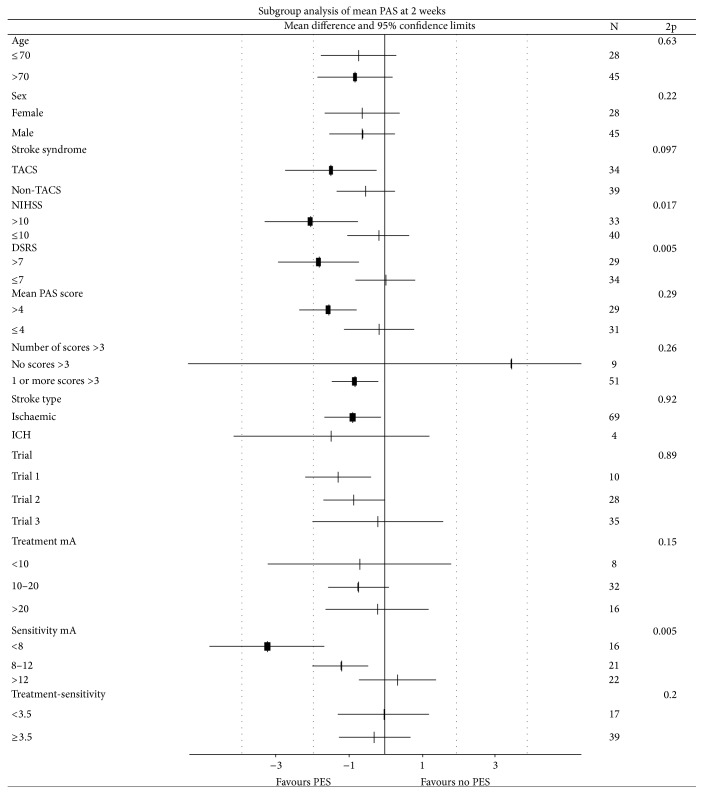
Mean penetration aspiration score, in subgroups: Age (≤70, >70), sex (female, male), stroke syndrome (non-TACS, TACS), stroke severity (NIHSS ≤10, >10), stroke type (IS, ICH); DSRS (≤7, >7), mean PAS (≤4, >4), number PAS >3 (0, >0), trial (1, 2, and 3), treatment current (<10, 10–20, and >20 mA), sensitivity current (<8, 8–12, and >12 mA), treatment-sensitivity current (<3.5, ≥3.5 mA). Interaction tests adjusted for trial, age, and baseline NIHSS and PAS.

In an adjusted analysis, patients randomised to PES had a significantly lower mean PAS score at 2 weeks by 0.9 points (of 9 points) than those assigned to no PES (Table 3); the effect of PES was consistent across the three trials (Supplemental Table II, Figure 1) Significant reductions in PAS were also seen when assessed using alternative analysis approaches: mean of the first 3 scores (difference 0.9 points), cumulative score across 6 boli (difference 6.0 points), and number of boli scoring >3 (Table 3). A trend to a lower worst bolus PAS score (i.e., worst PAS score across all boli) was also seen with PES and in unadjusted analyses using all statistical approaches. The effect of PES on mean PAS was assessed in prespecified subgroups (Figure 2); interactions were significant or had a trend (i.e., *p* < 0.1) for markers of stroke and dysphagia severity including baseline DSRS >7, TACS syndrome, and NIHSS >10; in each case, PES appeared to be effective in patients defined as being more severe. PES also appeared to be effective in patients who were sensitive to low stimulation currents. The effect of PES on PAS did not differ between the three trials (Figure 2).

### 3.5. Clinical Outcomes

**Figure 3:**
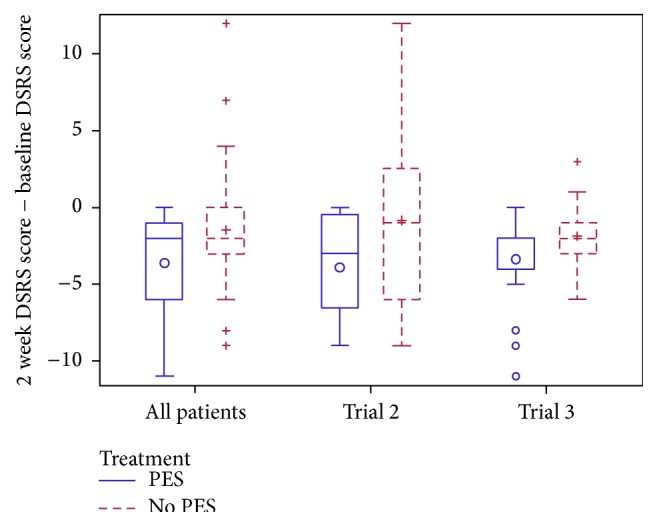
Box and whisker plot of change in dysphagia severity rating scale (DSRS) from baseline to two weeks, by source trial. Comparison of pharyngeal electrical stimulation (PES) versus no PES by multiple linear regression with adjustment, overall mean difference −1.7 (95% confidence interval −3.2, −0.1; *p* = 0.040); no difference between trials (*p* = 0.18).

Mean DSRS score was lower by 1.7 points (of 13 points) in patients randomised to PES versus no PES (Table 3) at 2 weeks; the effect of PES was consistent across the two trials that measured DSRS (Supplemental Table II, Figure 3). Additionally, the proportion of patients with clinical (functional) dysphagia at 2 weeks (DSRS >3) was lower in the group randomised to PES. The effect of PES on DSRS was assessed in prespecified subgroups (Supplemental Figure II); a significant interaction for treatment current was present with PES being more effective in patients who received 10–20 mA of stimulation. No differences in death, impairment (National Institutes of Health Stroke Scale), or respiratory tract infection (chest infection or pneumonia) were seen between the two groups. Patients randomised to PES tended to have a lower length of stay than those assigned to no PES (Table 3, Supplemental Figure III).

## 4. Discussion

Dysphagia is a common complication after stroke and is associated, independently, with a poor outcome. Although there are a number of interventions that show promise for treating dysphagia, none have definitive data [4]. Three trials have now been reported assessing the safety and efficacy of PES in patients with recent (subacute) stroke and dysphagia. In this individual patient data meta-analysis of data from these trials, PES was associated with reduced aspiration on videofluoroscopy (manifest as a lower penetration aspirations score) and a reduced proportion of patients with clinical dysphagia (defined as DSRS >3). Patients randomised to PES also had a trend to a reduced length of stay in hospital. When assessed in prespecified subgroups, PES appeared to be more effective in reducing radiological aspiration in patients with severe stroke, especially those with severe clinical dysphagia (DSRS >7), and in patients who were sensitive to low stimulation currents.

A number of interventions involving electrical stimulation have been tested in the rehabilitation of patients with stroke, including excitation of the vagus nerve, neck musculature (neuromuscular electrical stimulation) [17], pharynx (as here), sphenopalatine ganglion [18], and cranium (via transcranial direct current stimulation) [19]. In respect of dysphagia, electrical stimulation is thought to induce functional reorganisation of the swallowing cortex [7, 8] and this mechanism may explain the positive findings seen for PES with less aspiration and dysphagia. The observation that PES might be most effective in patients with severe dysphagia is plausible pathophysiologically and might allow treatment to be focused on patients who are more likely to respond. However, this observation is based on small numbers and needs replication. That PES might be associated with a shorter length of stay is encouraging from a health economic point of view and is likely to reflect less aspiration and dysphagia. The finding that low treatment levels of PES are most effective is counter-intuitive and needs confirmation in other studies; nevertheless, it may reflect that the pharynx is more sensitive and that this is a marker of potential recovery.

The optimal methods for analysing PAS and DSRS scores are unclear and a secondary aim of the present study was to test various statistical approaches. Mean PAS and the number of boli with PAS >3 were both statistically significant. Similarly, DSRS was significant when analysed both as continuous data and as the presence of clinical dysphagia (DSRS >3). Although binary outcomes are easier to explain to patients and healthcare staff, analyses using continuous or ordinal raw data are usually more efficient statistically, as seen previously for the mRS [20]. For both PAS and DSRS, analyses adjusted for baseline prognostic factors should be more sensitive to treatment change than those without adjustment, again as seen previously for the mRS [21].

The present systematic review and meta-analysis has a number of strengths. First, it used individual patient data (IPD) from the trials rather than summary/group data. Analyses based on IPD are considered to be the gold standard [22] and allow subgroup analyses to be performed, as done here. Second, data for all three identified and completed trials of PES were available thereby removing any bias through noninclusion of data. Third, aggregation of all available trials reduces the influence of any one study, better defines the point estimates of effects, and reduces the range of possible effect sizes.

Nevertheless, several caveats are present. First, the available data are small (3 trials, 73 patients) so the conclusions must be considered provisional. An ongoing larger study (STEPS trial), which will be twice this size of this analysis, will extend information on PES for poststroke dysphagia. Although other studies of PES have been performed, for example, in patients with stroke requiring ventilation, chronic stroke, and multiple sclerosis [23–25], there is no evidence that trials in acute and subacute stroke, as assessed here, were missed. Second, the trials were not double-blind although outcomes were assessed blinded to treatment; this design aspect is common in trials of medical devices and is difficult to overcome [26]. Third, although the mean baseline aspiration severity, as measured using the PAS, was relatively mild (mean 4.3), the PAS for the median worst swallow was 7 suggesting most patients were aspirating during VFS. Fourth, VFS might not be the optimal method for assessing aspiration (swallow results are variable both within and between boli, and patients with very severe stroke cannot participate in the process) and use of flexible endoscopic evaluation of swallowing (FEES) or even clinical judgment (as done here with the DSRS) might be preferable. Fifth, the size of effect seen with PES (improvements in PAS of 0.9 and DSRS 1.7) needs to be justified as being clinically important; both represent a >10% improvement in score, and both are associated with changes from severe to moderate, moderate to mild, or mild to no aspiration/dysphagia. Last, in two of the trials [9], PAS and DSRS were only measured at 2 weeks so that the effects of PES on longer-term aspiration and dysphagia are not clear and need to be studied in future trials.

In summary, PES appeared to reduce radiological aspiration and clinical dysphagia in patients with poststroke dysphagia, in three small trials. Larger trials are now needed to confirm these findings.

## Supplementary Material

Supplemental table I contains a list of studies identified by structured searches but which were excluded. The criteria for exclusion are also given.
Supplemental table II contains the mean PAS and DSRS before and after treatment in the PES and no PES groups in the three trials.
Supplemental figure I contains a flow diagram of the search for eligible trials.
Supplemental figure II contains a forest plot of a subgroup analysis of the Dysphagia Severity Rating Scale (DSRS) at 2 weeks.
Supplemental figure III contains a box and whisker plot of length of stay in hospital after randomisation, by source trial.

## Figures and Tables

**Table 1:** Design characteristics of completed trials of electrical pharyngeal stimulation in patients with recent stroke and dysphagia.

Trial	Jayasekeran-1 [9]	Jayasekeran-2 [9]	Vasant [10]
Year	2010	2010	2014
Participants (*N*)	10	28	35
Design	Dose comparison	Parallel group	Parallel group
Countries	1	1	1
Sites	1	2	3
PES sessions	1–3	3	3
Number of patients with PAS^†^	10	28	13
Bias [11]			
Random sequence	Low risk	Low risk	Low risk
Allocation sequence	Low risk	Low risk	Low risk
Blinding	High risk	High risk	High risk
Blinding of participants	Unclear risk	Unclear risk	Unclear risk
Blinding of outcome	Low risk	Low risk	Low risk
Incomplete outcome data	Low risk	High risk	High risk
Selective reporting	Low risk	Low risk	Low risk

^†^At baseline and 2 weeks.

Equipment: All studies used a transnasal catheter (3.2 mm diameter; Gaeltec Ltd., Isle of Skye, UK); this was connected to a preamplifier (CED 1902; Cambridge Electronic Design, Cambridge, UK) with signals processed (HumBug; Quest Scientific, North Vancouver, British Columbia, Canada) and recorded (Signal software, CED) running on a personal computer.

Funding:

Jayasekeran et al. 2010 [9]: Health Foundation, Medical Research Council.

Vasant et al. 2014 [10]: National Institutes of Health Research, Research for Patient Benefit.

**Table 2:** Baseline characteristics of participants. Data are number (%), median [interquartile range], or mean (standard deviation).

Trial	All	PES	No PES	Trial 1 [9]	Trial 2 [9]	Trial 3 [10]
Participants (*N*)	73	37	36	10	28	35
Age (years, mean, SD)	72.0 (11.8)	71.3 (13.4)	72.8 (9.9)	73.3 (11.9)	74.9 (9.7)	69.4 (12.9)
Sex (male, %)	45 (61.6)	24 (64.9)	21 (58.3)	5 (50.0)	19 (67.9)	21 (60.0)
mRS (/6)	3.0 (1.7) *n* = 43	3.1 (1.7) *n* = 21	2.9 (1.7) *n* = 22	0.1 (0.4) *n* = 8	NR	3.6 (1.0)
Stroke syndrome (%) [12]						
TACS	34 (46.6)	19 (51.4)	15 (41.7)	3 (30.0)	10 (35.7)	21 (60.0)
PACS	33 (45.2)	16 (43.2)	17 (47.2)	5 (50.0)	16 (57.1)	12 (34.3)
LACS	4 (5.5)	2 (5.4)	2 (5.6)	2 (20.0)	1 (3.6)	1 (2.9)
POCS	2 (2.7)	0 (0)	2 (5.6)	0 (0)	1 (3.6)	1 (2.9)
Type (%)						
Ischaemic	69 (94.5)	35 (94.6)	34 (94.4)	10 (100)	26 (92.9)	33 (94.3)
ICH	4 (5.5)	2 (5.4)	2 (5.6)	0 (0)	2 (7.1)	2 (5.7)
Severity, NIHSS (/42)	10.6 (5.8)	10.2 (5.9)	10.9 (5.8)	5.4 (4.2)	9.7 (4.1)	12.7 (6.4)
Side of lesion (%)						
Normal	12 (32.4) *n* = 37	7 (38.9) *n* = 18	5 (26.3) *n* = 19	5 (50.0)	—	7 (25.9) *n* = 27
Left	6 (16.2)	3 (16.7)	3 (15.8)	1 (10.0)	—	5 (18.5)
Right	15 (40.5)	6 (33.3)	9 (47.4)	4 (40.0)	—	11 (40.7)
Bilateral	4 (10.8)	2 (11.2)	2 (10.5)	0 (0)	—	4 (14.8)
Feeding, enteral (%)	36 (49.3)	18 (48.6)	18 (50.0)	2 (20.0)	11 (39.3)	23 (65.7)
DSRS (/12)	6.7 (4.3) *n* = 63	7.1 (4.2) *n* = 33	6.3 (4.4) *n* = 30	—	6.0 (4.8)	7.3 (3.8)
Median [IQR]	6.0 [9.0] *n* = 63	8.0 [8.0] *n* = 33	5.5 [9.0] *n* = 30	—	5.5 [10.5]	7.0 [8.0]
= 12 (%)	17 (27.0) *n* = 63	9 (27.3) *n* = 33	8 (26.7) *n* = 30	—	7 (25.0)	10 (28.6)
Weight (kg)	76.9 (23.6) *n* = 41	75.2 (24.8) *n* = 20	78.5 (22.9) *n* = 21	78.0 (24.8) *n* = 6	—	76.7 (23.8)
PAS (/8)	4.3 (1.8) *n* = 60	4.6 (2.0) *n* = 32	3.9 (1.6) *n* = 28	4.5 (0.8)	4.3 (1.9)	4.2 (2.2) *n* = 22
Mean of first 3	4.3 (2.2) *n* = 60	4.7 (2.5) *n* = 32	3.9 (1.8) *n* = 28	4.9 (1.8)	4.1 (2.1)	4.3 (2.5) *n* = 22
Number of boli >3	3.2 (2.1) *n* = 60	3.6 (2.2) *n* = 32	2.7 (2.0) *n* = 28	3.5 (1.5)	3.4 (2.3)	2.6 (2.1) *n* = 22
Any bolus >3 (%)	51 (85.0) *n* = 60	28 (87.5) *n* = 32	23 (82.1) *n* = 28	9 (90.0)	23 (82.1)	19 (86.4) *n* = 22
Worst [median, IQR]	7.0 [3.0] *n* = 60	7.5 [3.0] *n* = 32	6.0 [3.5] *n* = 28	8.0 [3.0]	7.0 [3.0]	6.0 [3.0] *n* = 22
Cumulative [/48]	25.0 [14.5] *n* = 56	28.1 [17.0] *n* = 30	23.5 [11.0] *n* = 26	27.0 [5.0]	26.1 [16.0]	20.5 [19] *n* = 18
TAR (days)	15.1 (8.5)	14.8 (8.5)	15.5 (8.7)	13.2 (7.3)	15.0 (7.7)	15.8 (9.6)
Electrical current (mA)						
Sensitivity	11.4 (5.6) *n* = 59	10.4 (5.3) *n* = 33	12.8 (5.8) *n* = 26	7.9 (3.2) *n* = 4	10.3 (4.4) *n* = 26	12.9 (6.4) *n* = 29
Treatment	16.8 (6.6) *n* = 56	15.5 (5.7) *n* = 30	18.4 (7.2) *n* = 26	12.3 (4.3) *n* = 4	15.3 (5.2) *n* = 23	18.6 (7.3) *n* = 29

DSRS: dysphagia severity rating scale; ICH: intracerebral haemorrhage; LACS: lacunar syndrome; mRS: modified Rankin Scale; NIHSS: National Institutes of Health Stroke Scale; NR: not recorded; PACS: partial anterior circulation syndrome; PAS: penetration aspiration score; POCS; posterior circulation syndrome; SD: standard deviation; TACS: total anterior circulation syndrome; TAR: time admission-randomisation.

**Table 3:** Comparison of outcomes at 2 weeks and discharge from hospital in 73 patients across three trials by treatment assignment (pharyngeal electrical stimulation versus sham/placebo). Data are number (%), median [interquartile range], or mean (standard deviation). Comparison by binary logistic regression (BLR), ordinal logistic regression (OLR), or multiple regression (MR), with 95% confidence intervals. Adjustment: full model is adjusted for trial, relevant baseline score, age, and impairment (NIHSS); partial model is adjusted for trial only.

	Trials/patients	PES	Sham	Difference/odds ratio (95% CI)	2*p*	Difference/odds ratio (95% CI)	2*p*
Statistical model		37	36	Adjusted, full		Adjustment, trial	
*2 weeks*							
PAS (/8)	3/55	3.4 (1.7)	4.1 (1.7)	**−0.9 (−1.7**,** −0.1)**	**0.020**	−0.7 (−1.6, 0.2)	0.13
Mean of first three	3/55	3.2 (1.7)	3.9 (1.9)	**−0.9 (−1.7**,** 0.0)**	**0.044**	−0.6 (−1.5, 0.3)	0.19
Cumulative (/48)	3/54	19.3 (9.6)	24.4 (10.4)	**−6.0 (−10.7**,** −1.4)**	**0.011**	−4.7 (−9.8, 0.5)	0.078
Number of boli >3	3/55	2.2 (2.1)	3.3 (2.0)	**−1.4 (−2.2**,** −0.6)**	**<0.001**	−1.0 (−2.1, 0.0)	0.055
Any bolus >3 (%)	3/55	22 (75.9)	24 (92.3)	**0.05 (0**,** 0.96)**	**0.047**	0.26 (0.05, 1.49)	0.13
Worst [/8]	3/55	5 [2]	6 [3]	0.37 (0.11, 1.18)	0.092	0.7 (0.27, 1.81)	0.46
DSRS (/12)	2/63	3.5 (3.8)	4.9 (4.4)	**−1.7 (−3.2**,** −0.1)**	**0.040**	−1.3 (−3.3, 0.7)	0.19
Score >3 (%)	2/63	10 (30.3)	16 (53.33)	**0.25 (0.07**,** 0.89)**	**0.032**	0.39 (0.14, 1.10)	0.075
Score >7 (%)	2/63	6 (18.2)	7 (23.3)	0.54 (0.12, 2.36)	0.41	0.79 (0.23, 2.78)	0.71
NIHSS (/42)	2/44	7.9 (5.6)	9.1 (8.5)	0.4 (−2.5, 3.4)	0.77	−1.1 (−5.2, 3.1)	0.62
Died (%)	1/35	0 (0)	1 (5.6)	—	—	—	—
RTI (%)	2/63	4 (12.1)	4 (13.3)	—	—	0.83 (0.18, 3.74)	0.81
*Discharge*							
Hospital stay (days)	3/73	50.2 (25.3)	71.2 (60.4)	−16.2 (−36.2, 3.7)	0.11	−18.5 (−39.0, 1.9)	0.075
Died, in hospital (%)	1/35	0 (0)	1 (5.6)	—	—	—	—
Died, end of trial (%)	1/35	1 (5.9)	1 (5.6)	1.2 (0.1, 25.8)	0.91	1.1 (0.1, 18.5)	0.97

Scores for death: DSRS = 13, NIHSS = 43, and PAS = 9.

DSRS: dysphagia severity rating scale; NIHSS: National Institutes of Health Stroke Scale; PAS: penetration aspiration score; RTI: respiratory tract infection (chest infection or pneumonia).
