# Supplementary material for: Pharyngeal Electrical Stimulation for Treatment of Poststroke Dysphagia: Individual Patient Data Meta-Analysis of Randomised Controlled Trials
Source: Stroke Res Treat. 2015 Nov 24;2015:429053. doi: 10.1155/2015/429053 (PMC4672134; doi:10.1155/2015/429053)
Supplement: Supplementary file 1 — Supplemental table I contains a list of studies identified by structured searches but which were excluded. The criteria for exclusion are also given. Supplemental table II contains the mean PAS and DSRS before and after treatment in the PES and no PES groups in the three trials. Supplemental figure I contains a flow diagram of the search for eligible trials. Supplemental figure II contains a forest plot of a subgroup analysis of the Dysphagia Severity Rating Scale (DSRS) at 2 weeks. Supplemental figure III contains a box and whisker plot of length of stay in hospital after randomisation, by source trial. [file 429053.f1.pdf]

SUPPLEMENTAL MATERIAL

Supplemental Table I. Studies that were identified in structured searches but were excluded, and criteria for exclusion.

Study Reference	Reason for Exclusion
Archer 2013 [1]	• Non-RCT
Bulow 2008 [2]	• Non-PES study
Burnett 2005 [3]	• Non-PES study<br>• Non-RCT<br>• Non-stroke
Byung-Mo 2010 [4]	• Non-RCT
Clark 2009 [5]	• Non-RCT<br>• Systematic review
Heijnen 2012 [6]	• Non-PES study<br>• Non-stoke
Jayasekeran 2011 [7]	• Non-RCT<br>• Non-stroke
Lim 2009 [8]	• Non-PES study
Long 2013 [9]	• Non-PES study<br>• Non-stroke
Ludlow [10]	• Non-RCT
Michou 2014 [11]	• Non-RCT<br>• Chronic stroke
Restivo 2013 [12]	• Non-stoke
Restivo 2014 [13]	• Non-stroke<br>• Not PES
Suntrup 2013 [14]	• Non-RCT
Rofes 2013 [15]	• Non-RCT
Rogus-Pulia 2013 [16]	• Non-RCT<br>• Review
Speyer 2010 [17]	• Non-RCT<br>• Systematic review
STEPS 2015 http://www.phagenesis.com/clinical/STEPS-study	• Ongoing RCT
Tan 2013 [18]	• Not an PES RCT<br>• Multiple aetiologies

PES: Pharyngeal electrical stimulation; RCT: randomised controlled trial

Supplemental Table II. Mean penetration aspiration score (PAS) and dysphagia severity rating scale (DSRS) before and after treatment in the active and sham groups in the 3 trials. Patients with both baseline and 2 week mean PAS are included. Comparison by t test.

	Timing	PAS			DSRS		
		Trial 1 19	Trial 2 19	Trial 3 20	Trial 1 19	Trial 2 19	Trial 3 20
Active	Before	4.8	4.6	4.5	-	6.4	7.8
	After	3.7	3.2	2.6	-	2.5	4.4
	Difference	-1.0	-1.4	-1.9	-	-3.9	-3.4
Sham	Before	4.3	3.9	3.9	-	5.6	6.8
	After	4.8	3.8	4.3	-	4.8	5.0
	Difference	+0.5	-0.1	+0.4	-	-0.8	-1.8
Active-sham	Difference	-1.5	-1.3	-2.3	-	-3.0	-1.5
	p	0.017	0.061	0.22	-	0.11	0.11

Supplemental Figure I. Flow diagram of search for trials

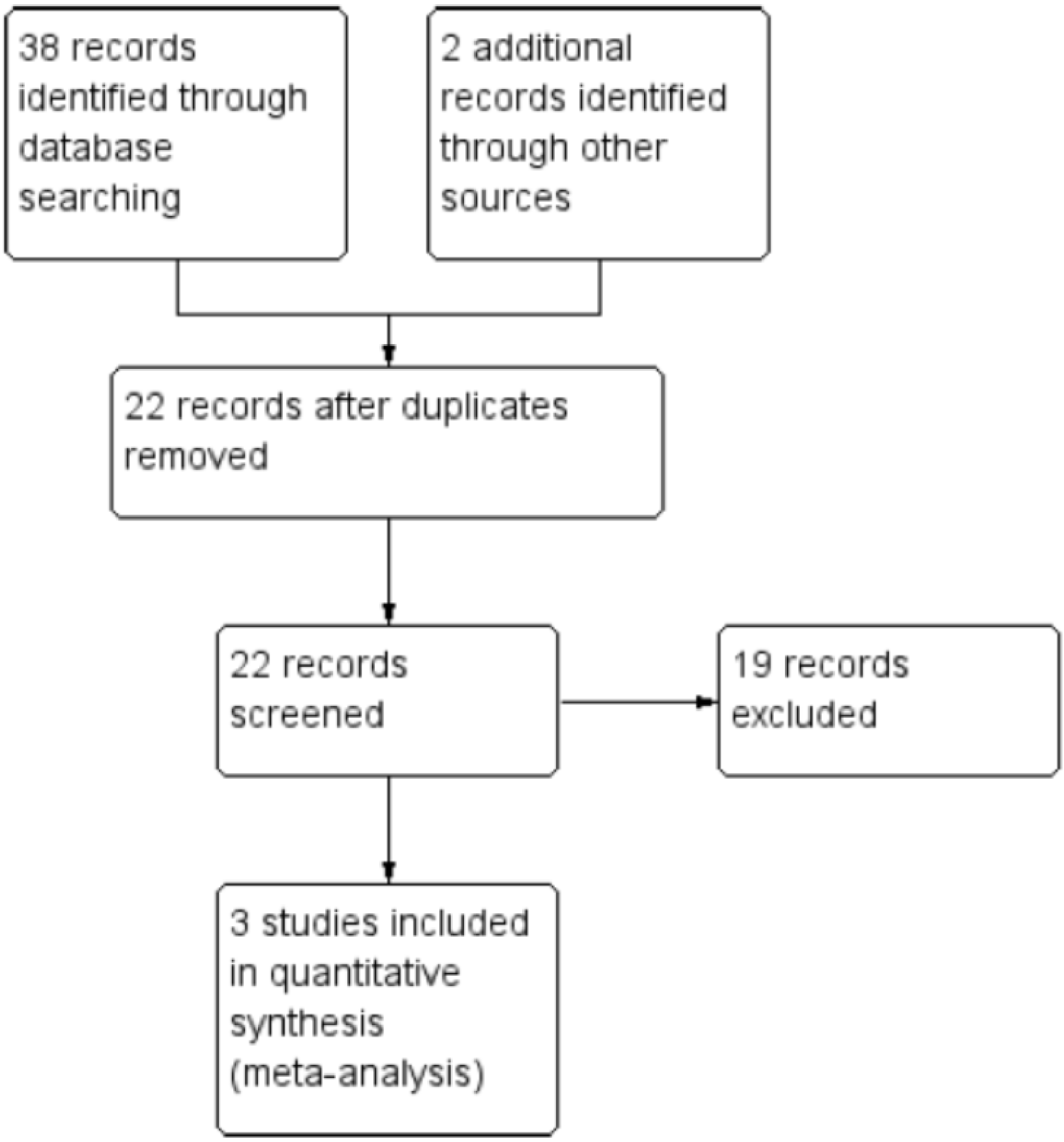

Supplemental Figure II. Dysphagia Severity Rating Scale, in subgroups (as for penetration aspiration score in Figure 2)

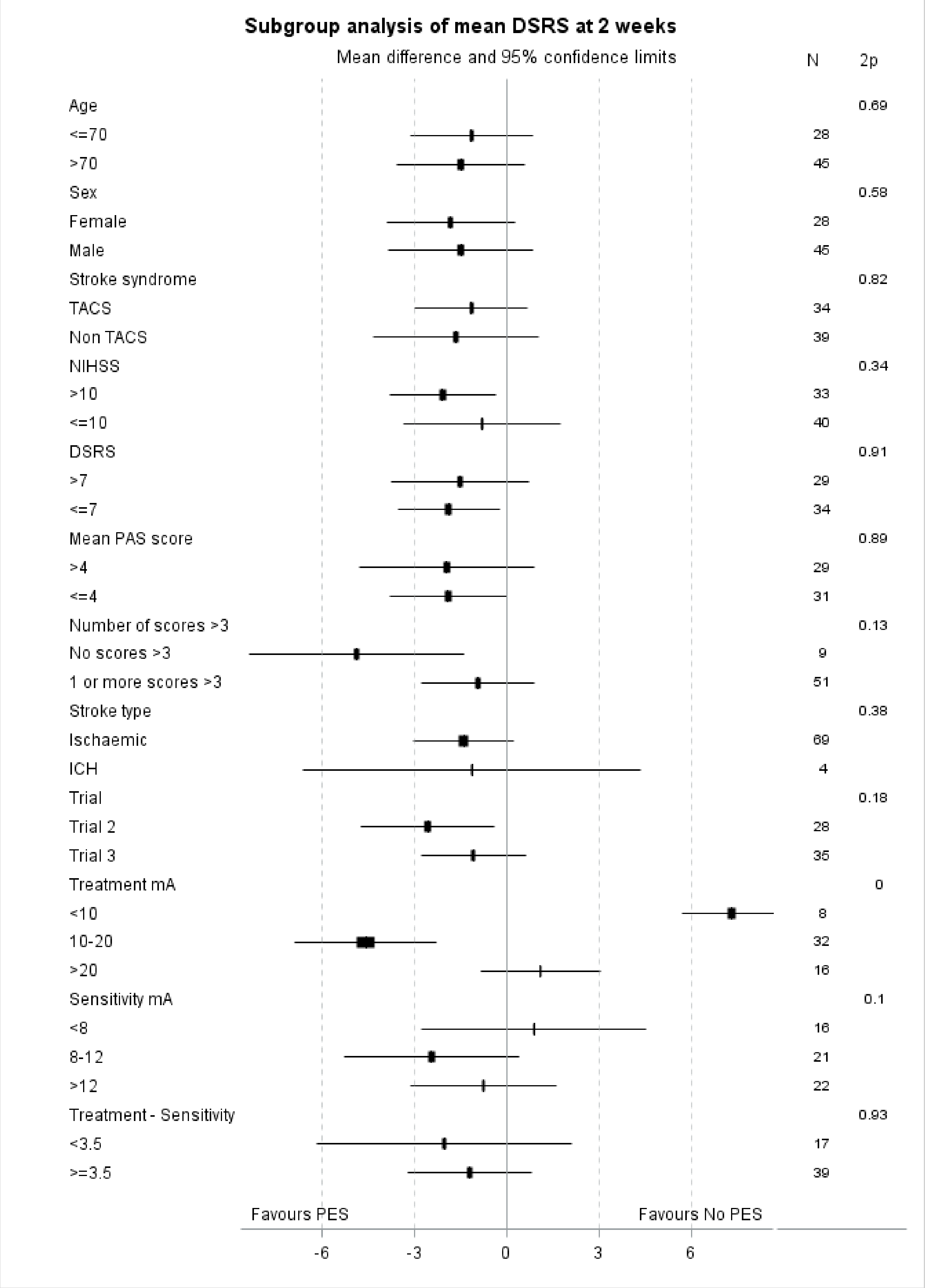

Supplemental Figure III. Box and whisker plot of length in stay in hospital after randomisation, by source trial. Comparison of PES versus no PES by multiple linear regression with adjustment, overall mean difference -16.2 (95% CI -36.2, 3.7; p=0.11). Note: one patient randomised to sham PES with length of stay of 365 days excluded to make figure more clear.

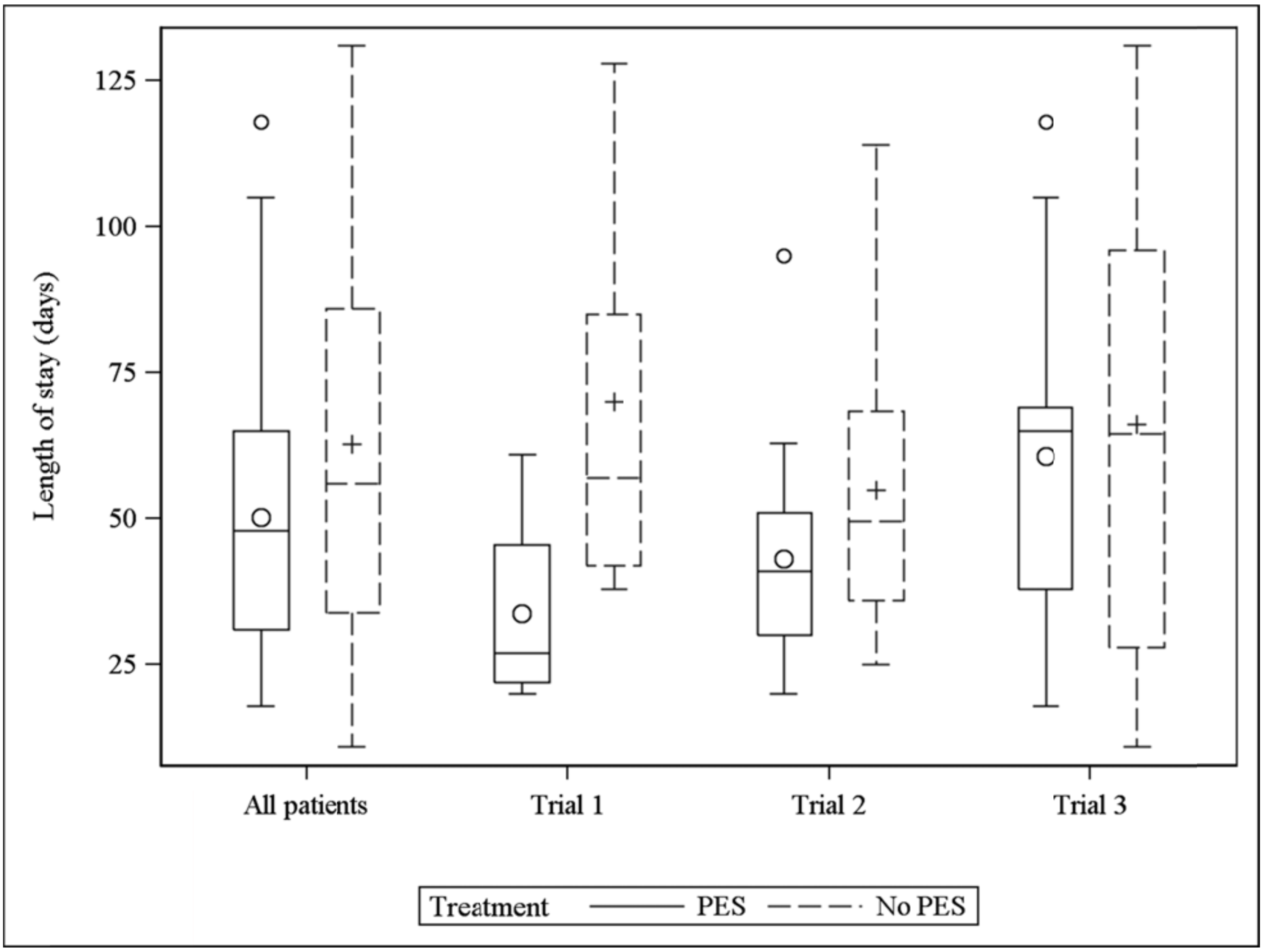

Supplemental References

1. Archer S, Wellwood I, Smith C, Newham D. Dysphagia therapy in stroke: A survey of speech and and language therapists. *International Journal of Language and Communication Disorders*. 2013;48:283-296
2. Bülow M, Speyer R, Baijens L, Woisard V, Ekberg O. Neuromuscular electrical stimulation (nmes) in stroke patients with oral and pharyngeal dysfunction. *Dysphagia*. 2008;23:302-309
3. Burnett T, Mann E, Stoklosa J, Ludlow C. Self-triggered functional electrical stimulation during swallowing. *Journal of Neurophysiology*. 2005;94:4011-4018
4. Byung-Mo O, Nam-Jong P. *Management of swallowing difficulty in pharyngeal diseases*. Nova Science Pulblishers, Inc.; 2010.
5. Clark H, Lazarus C, Arvedson J, Schooling T, Frymark T. Evidence-based systematic review: Effects of neuromuscular eletrical stimulation on swallowing and neural activation. *American Journal of Speech and Langualge Pathology*. 2009;18:361-375
6. Heijen B, Speyer R, Baijens L, Bogaardt H. Neuromuscular electrical stimulation versus traditional therapy in patients with parkinson's disease and oropharyngeal dysphagia: Effects on quality of life. *Dysphagia*. 2012;27:336-345
7. Jayasekeran V, Pendleton N, Holland G, Payton A, Jefferson S, Michou E, et al. Val66met in brain-derived neurotrophic factor affects stimulus-induced plasticity in the human pharyngeal motor cortex. *Gastroenterology*. 2011;141:827-836 e821-823
8. Lim KB, Lee HJ, Lim SS, Choi YI. Neuromuscular electrical and thermal-tactile stimulation for dysphagia caused by stroke: A randomized controlled trial. *J Rehabil Med*. 2009;41:174-178
9. Long Y, Wu X. A randomized controlled trial of combination therapy of neuromuscular electrical stimulation and balloon dilatation in the treatment of radiation-induced dysphagia in nasopharyngeal carcinoma patients. *Disability and rehabilitation*. 2013;35:450-454
10. Ludlow C. Electrical neuromuscular stimulation in dysphagia: Current status. *Current Opinion in Otolaryngology & Head and Neck Surgery*. 2010;18:159-164
11. Michou E, Mistry S, Jefferson S, Tyrrell P, Hamdy S. Characterizing the mechanisms of central and peripheral forms of neurostimulation in chronic dysphagic stroke patients. *Brain Stimulation*. 2014;7:66-73
12. Restivo D, Casabona A, Centonze D, Marchese-Ragona R, Maimone D, Pavone A. Pharyngeal electrical stimulation for dysphagia associated with multiple sclerosis: A pilot study. *Brain Stimulation*. 2013;6:418-423
13. Restivo D, Casabono A, Marchese-Ragona R. Transcranial direct current stimulation (tdcs) for dysphagia associated to multiple sclerosis. *Clinical Neurophysiology*. 2014;125:S205
14. Suntrup S. Motor cortical reorganization in dysphagia following stroke. . *Klinische Neurophysiologie*. 2013;44:247-256
15. Rofes L, Vilardell N, Clave P. Post-stroke dysphagia: Progress at last. *Neurogastroenterology and Motility*. 2013;25:278-282

16. Rogus-Pulia N, Robbins J. Approaches to the rehabilitation of dysphagia in acute poststroke patients. *Seminars in Speech and Language*. 2013;34:154-169
17. Speyer R, Baijens L, Heijen M, Zwijnenberg I. Effects of therapy in oropharyngeal dysphagia by speech and language therapists: A systematic review. *Dysphagia*. 2010;25:40-65
18. Tan C, Liu Y, Li W, Liu J, Chen L. Transcutaneous neuromuscular electrical stimulation can improve swallowing function in patients with dysphagia caused by non-stroke diseases: A meta-analysis. *Journal of Oral Rehabilitation*. 2013;40:472-480
19. Jayasekeran V, Singh S, Tyrrell P, Michou E, Jefferson S, Mistry S, et al. Adjunctive functional pharyngeal electrical stimulation reverses swallowing disability after brain lesions. *Gastroenterology*. 2010;138:1737-1746
20. Vasant D, Michou E, Tyrrell P, Jayasekeran V, Mistry S, O'Leary N, et al. Oc-063 pharyngeal electrical stimulation (pes) in dysphagia post-acute stroke: A double blind, randomised trial. *Gut*. 2014;63
